# Redo-Transcatheter Aortic Valve Implantation (Redo-TAVI)—Pilot Study from Multicentre Nationwide Registry

**DOI:** 10.3390/jcm14228078

**Published:** 2025-11-14

**Authors:** Szymon Jonik, Maciej Mazurek, Bartosz Rymuza, Jan Jankowski, Maciej Dąbrowski, Rafał Wolny, Piotr Chodór, Krzysztof Wilczek, Wojciech Fil, Krzysztof Milewski, Marcin Protasiewicz, Krzysztof Ściborski, Agnieszka Kapłon-Cieślicka, Alicja Skrobucha, Michał Hawranek, Piotr Scisło, Radosław Wilimski, Janusz Kochman, Marcin Grabowski, Marek Grygier, Adam Witkowski, Zenon Huczek

**Affiliations:** 1 1st Department of Cardiology, Medical University of Warsaw, 02-097 Warsaw, Poland; maciej.j.mazurek@gmail.com (M.M.); bartosz.rymuza@gmail.com (B.R.); agnieszka.kaplon@gmail.com (A.K.-C.); alicja.skrobucha@gmail.com (A.S.); scislo@wum.edu.pl (P.S.); janusz.kochman@wum.edu.pl (J.K.); marcin.grabowski@wum.edu.pl (M.G.); zhuczek@wp.pl (Z.H.); 2 Department of Interventional Cardiology and Angiology, National Institute of Cardiology, 04-628 Warsaw, Poland; macidabro@gmail.com (M.D.); rafal.wolny@gmail.com (R.W.); witkowski@hbz.pl (A.W.); 3 Department of Cardiology and Electrotherapy, Faculty of Medical Science in Zabrze, Medical University of Silesia, 41-808 Katowice, Poland; chodor_piotr@go2.pl (P.C.); kwilczek@sum.edu.pl (K.W.); 4 Polish-American Heart Clinic, 43-316 Bielsko-Biała, Poland; wojtek_fil@op.pl (W.F.); kpmilewski@gmail.com (K.M.); 5 Department of Cardiology, Wroclaw Medical University, 50-368 Wroclaw, Poland; mprot@poczta.onet.pl; 6 Jan Mikulicz Radecki University Hospital, 50367 Wroclaw, Poland; 7 Department of Cardiology, 4th Military Hospital, 50-981 Wroclaw, Poland; k.sciborski@op.pl; 8 3rd Department of Cardiology, Faculty of Medical Sciences in Zabrze, Medical University of Silesia, 40-752 Katowice, Poland; mhawranek@poczta.fm; 9 Department of Cardiothoracic Surgery and Transplantology, Medical University of Warsaw, 02-097 Warsaw, Poland; rwilimski@gmail.com; 10 Department of Cardiology, Poznan University of Medical Sciences, 61-866 Poznan, Poland; marek.grygier@skpp.edu.pl

**Keywords:** redo-TAVI, index TAVI, valve-in-valve procedure, bioprosthesis failure, procedure failure

## Abstract

**Objectives**: The aim of this study is to evaluate the safety and efficacy of repeat transcatheter aortic valve implantation (redo-TAVI) in the polish population. **Methods**: In this multicentre nationwide registry (ClinicalTrials.gov identifier, NCT03361046), we provide characteristics, periprocedural variables and long-term outcomes of high-risk patients who underwent redo-TAVI. **Results**: The mean age among 32 individuals who underwent redo-TAVI was 75 ± 13 years, and 62.5% were male. The mean time from index TAVI to redo-TAVI was 4.7 ± 3.5 years, with failed procedures (up to 1 year) occurring in 7 (21.9%) and failed transcatheter heart valve (THV, beyond 1 year) in the remaining majority of the 25 (78.1%) patients. Computed tomography-based native bicuspid aortic anatomy was found frequently in 37.5% of cases (58.3% in failed procedures and 41.7% in failed THV). The mean failed THV size was large (27.7 ± 3 mm) and predominantly presenting with pure regurgitation (59.4%). In more than two-thirds (68.7%), balloon-expandable or self-expandable THV was the most common strategy of redo-TAVI. None or mild regurgitation was found in 90.6%, and the mean transvalvular gradient was 13.1 ± 5.5 mmHg, with only three cases with >20 mmHg of the residual gradient (9.4%). Peri-procedural and 30-day complications were low, and cardiovascular and all-cause mortality at 1 year was 9.4 and 15.6%, respectively. There was a relatively high incidence of non-procedural stroke after redo-TAVI (*n* = 5, 15.6%), with all cases observed after 30 days. **Conclusions**: Initial data of redo-TAVI in Poland suggest that the procedure is safe and characterized by favourable efficacy and low rates of short-term adverse outcomes. A high frequency of baseline native bicuspid anatomy and late stroke occurrence after the redo-procedure warrants further investigation in larger cohorts.

## 1. Introduction

According to current European guidelines, transcatheter aortic valve implantation (TAVI) provides effective treatment for native severe symptomatic aortic stenosis (AS) in individuals who are 75 years or older [1]. Recent encouraging results of TAVI application in younger populations with AS, coupled with overall progressively longer lifetime expectancy, will most probably result in the need for repeat transcatheter treatment (redo-TAVI) due to transcatheter valve (THV) failure in some patients in the near future [2]. Previous retrospective international registries estimated the frequency of redo-procedures to be very low, less than 0.5% of all TAVI procedures; however recent analyses from North America, where guidelines allow for TAVI procedures at a lower age (65 years or older), predict a steep annual increase, with an estimated number of redo-TAVI procedures as high as 30 thousand per year in a decade from now [3]. Another issue requiring evaluation is the population of patients with a bicuspid aortic valve (BAV) undergoing the TAVI procedure. Generally, according to current guidelines, conventional surgical aortic valve replacement (SAVR) has historically been the recommended treatment option for patients with a bicuspid aortic valve anatomy. However, considering the highly burdened population, as well as the increased availability of percutaneous techniques and their significantly less invasive nature, TAVI is gaining popularity for BAV stenosis [1,2]. In the present paper, on the basis of a nationwide multicenter prospective registry, we present the first preliminary experience of redo-TAVI in Poland. A pilot study such as this one may provide very valuable date for future trials and analyses.

## 2. Materials and Methods

The Polish Transcatheter Aortic Valve-in-Valve Implantation (ViV-TAVI) Registry (ClinicalTrials.gov identifier, NCT03361046) was initiated on 1 January 2018 and designed to collect data of all patients who underwent ViV-TAVI (including any type of surgical THV [redo-TAVI]) [4]. Procedures performed in a position other than the aortic valve were excluded. A decision to refer a patient for redo-TAVI was made by a Heart Team at each of the 7 centres in Poland reporting redo-TAVI procedures. Anonymized data were collected retrospectively since 2013 (1st redo-procedure in Poland) and then prospectively after the start of the registry. Data collection was completed in January 2025. Investigators at contributing centres reported the data using a dedicated online case report form. The reported data were monitored for inconsistencies.

First TAVI in native AS is referred to as “index TAVI”, and repeat TAVI is referred to as “redo-TAVI”. “Failed THV” was defined as valve degeneration (mostly structural but also due to previous endocarditis, thrombosis or both), and a “failed TAVI procedure” was defined as suboptimal positioning (too low/too high) or mal-sizing (under or oversizing, asymmetrical expansion, etc.) of the index THV. As previously proposed, an arbitrary cut-off time was adopted: for a failed TAVI procedure, the cut-off time was when redo-TAVI was performed within 1 year of the index procedure, and for failed THV, it was when redo-TAVI was performed beyond 1 year [3]. We excluded patients with bail-out implantation of a second THV during the same procedure. For echocardiography before redo-TAVI, index THV dysfunction could be classified as pure stenosis, pure regurgitation or mixed etiology (at a least moderate degree of stenosis [mean pressure gradient > 20 mmHg] and concomitant regurgitation [at least grade 3 or 4]). Surgical risk was evaluated using the online calculator for the STS (Society of Thoracic Surgeons) score. The severity of heart failure symptoms was assessed using New York Heart Association (NYHA) classifications. Procedural data included the type and size of the index THV, the type and size of redo-THV, pre- or post-dilatation and the need for coronary protection. Echocardiographic and 1-year clinical follow-ups were performed. Informed written consent was obtained from all subjects involved in the study.

### Statistical Analysis

All analyses were performed in accordance with the relevant guidelines and regulations. The PQStat software (version 1.6.6, PQStat, Poznań, Poland) was used for statistical analysis. The normality of distribution for continuous variables was confirmed with the Shapiro–Wilk test. Categorical data were expressed as counts and percentages, while continuous data were presented as the mean and standard deviation. Overall mortality was analyzed as a separate dependent variable using the Cox model. The Variance Inflation Factor (VIF) was used to detect multicollinearity among predictor variables. Variables with VIF values less than 5 are considered acceptable.

## 3. Results

### 3.1. Baseline Characteristics Before Redo-Procedures and Index TAVI Details

In seven participating high-volume TAVI centres, a total of 32 redo-TAVI procedures were performed between January 2013 and January 2024, with almost half of the procedures (*n* = 15) reported in the last 2 years.

**Figure 1:**
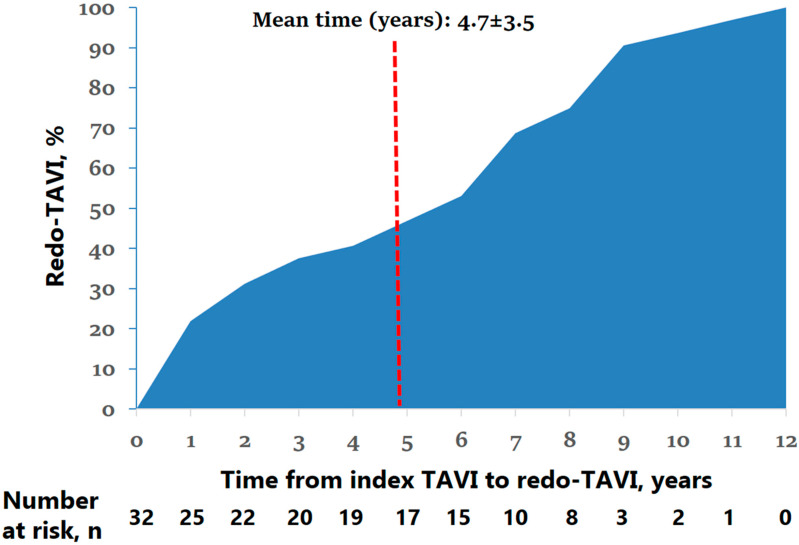
Mean time from index TAVI to redo-TAVI, years.

Baseline characteristics and index TAVI details are presented in Table 1, Table 2 and Table 3. The mean age of the studied population was 75 ± 13 years, and 62.5% were male. Generally, patients were categorized as high-risk; e.g., one-third had a history of myocardial infarction; a quarter presented after coronary artery bypass grafting; almost half had atrial fibrillation; and half had a history of percutaneous coronary intervention. The mean time from index TAVI to redo-TAVI was 4.7 ± 3.5 years (shortest was 14 days, and longest was 11.1 years), with failed procedures (up to 1 year) occurring in 7 (21.9%) and failed THV (beyond 1 year) occurring in the remaining majority of the 25 (78.1%) patients (Figure 1 and Table 4). Index THV endocarditis or thrombosis was found in five patients (15.6%). In the majority of cases (84.4%), index THV was self-expandable with a mean size of 27.7 ± 3 mm. In almost two-thirds of patients, dual antiplatelet therapy was prescribed at discharge. The predominant mechanism of failure was pure regurgitation (59.4%), followed by pure stenosis and mixed failure. Interestingly, based on CT analysis of index valve stenosis, in 37.5%, bicuspid anatomy was found (predominantly type 1; 42.8% in failed procedure and 36% in failed THV).

### 3.2. Redo-TAVI Procedural Characteristics and 1-Year Outcomes

Key periprocedural characteristics and outcomes are presented in Table 4 and Table 5. Most frequently, balloon-expandable prosthesis THV was implanted during redo-TAVI (75%), and in the majority of the remaining patients, a combination of self-expandable systems in failed balloon-expandable systems was reported (18.9%). The same type of valve was only implanted into the same type of THV in four cases (*n* = 3 for self-expandable in self-expandable; *n* = 1 for balloon-expandable in balloon-expandable). The mean redo-THV label size was 25 ± 3 mm. In 12.5% of patients, a high risk of coronary artery occlusion was identified based on valve-to-coronary distances in CT scans, and leaflet laceration or chimney stenting was performed to prevent acute closure of the coronary ostia.

**Figure 2:**
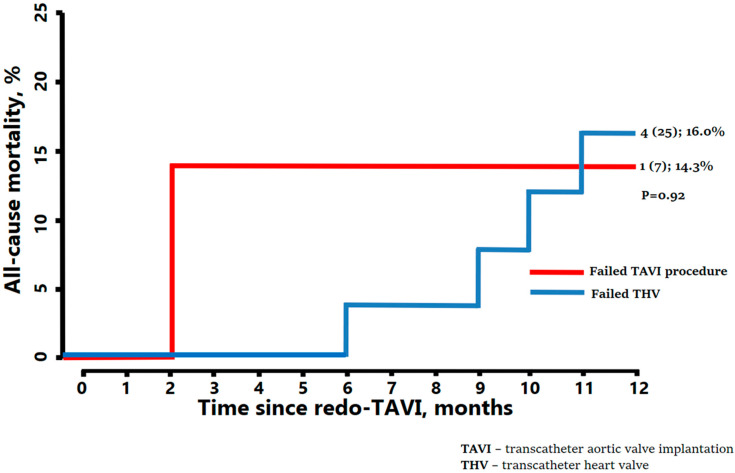
All-cause mortality after redo-TAVI (months).

The mean transvalvular gradient after redo-TAVI was 13.1 ± 5.5 mmHg, and the mean pressure gradient was only greater than 20 mmHg in three patients. In more than 90% of patients, clinical improvement in symptoms (NYHA I or II) was observed immediately after the procedure. Conduction disturbances were rare and limited to the occurrence of a new left bundle branch block, with no need for permanent pacemaker implantation (PPI). In short-term follow-ups, up to 30 days, there were no deaths or strokes, and one patient required surgery due to severe residual aortic regurgitation. One year after the redo-procedure, five patients died (15.6%; 1/7 [14.3%] in the failed procedure group and 4/25 [16%] in the failed THV group, *p* = 0.92), and of those, three were from cardiovascular causes [Figure 2]. In the period between 30 days and 1 year, there were five non-procedural strokes (15.6%) reported, including two that were disabling. Two of those patients had a history of atrial fibrillation and subsequent oral anticoagulation prescribed at discharge, while one received dual antiplatelet therapy, and the remaining two were prescribed single antiplatelet therapy.

### 3.3. Multivariable Cox Proportional Hazard for the Primary Endpoint

Among parameters from clinical, periprocedural and echocardiographic variables, nine showed a significant relationship with overall mortality: (1) age-per-year increase [HR (95% CI)]: 1.06 (1.01–1.33), *p* = 0.02; (2) diabetes mellitus [HR (95% CI)]: 1.88 (1.12–3.95), *p* = 0.04; (3) COPD [HR (95% CI)]: 2.34 (1.35–4.13), *p* = 0.01; (4) prior stroke or a transient ischemic attack [HR (95% CI)]: 2.64 (1.53–4.66), *p* < 0.001; (5) chronic kidney disease [HR (95% CI)]: 1.96 (1.48–3.81), *p* = 0.02; (6) bicuspid anatomy [HR (95% CI)]: 3.01 (2.09–5.85), *p* < 0.001; (7) perivalvular leak moderate/severe (assessed by echocardiography) [HR (95% CI)]: 2.97 (1.97–5.77), *p* < 0.001; (8) baseline mechanism of failure—stenosis [HR (95% CI)]: 1.82 (1.44–3.14), *p* = 0.01; and (9) a history of index THV endocarditis [HR (95% CI)]: 1.99 (1.55–3.12), *p* = 0.01.

## 4. Discussion

The number of repeat TAVI procedures, although still very low, is expected to rise with the growing implementation of TAVI among low-risk individuals with AS [5]. Even greater growth is expected if lower ages (<75 years) were included in the upcoming European guidelines [6]. Given the natural history of any bioprostheses, as well as those transcatheter ones, a scenario of redo-TAVI would probably become the treatment of choice for degenerated THVs, especially with regard to the substantial mortality risk observed in SAVR when performed after TAVI [7]. Therefore, it is highly important to evaluate characteristics and first experiences of redo-TAVI procedures in Poland.

In the Polish cohort, a majority of patients treated with redo-TAVI (with the previously used time cut-off of 1 year) had failed THV rather than a failed TAVI procedure (as the result of THV misplacement or missizing was 78.1% vs. 21.9%, respectively). Given that the mean label size of failed THVs was large and that the predominant mechanism of failure in almost 60% was pure regurgitation, implantation of redo-THV was rarely associated with elevated gradients (mean echocardiographic gradient > 20 mmHg in only 9.4%). This is most likely the result of the predominantly self-expanding THV market share in Poland in the analyzed period, and with greater representation of balloon-expanding smaller devices, this could be somewhat different [8]. An interesting finding of the present registry is definitely the relatively high percentage of native CT-based bicuspid anatomy, not only in the failed procedure individuals (58.3%) but also in the failed THV cohort (41.7%). This is a much higher incidence of bicuspid AS compared to available data on its observed frequency among native TAVI procedures [9]. So far, SAVR has been the traditional and well-established approach for aortic valve replacement, and it has a strong track record of success in patients with bicuspid valves. However, TAVI offers a less invasive option (especially for highly burdened patients without other conditions that require surgery, such as an enlarged aortic root, complex coronary artery disease or severe mitral regurgitation) for treating bicuspid aortic stenosis. With advancements in valve technology and increased physician expertise, TAVI is being considered for more BAV patients. The introduction of third-generation improved THVs with external wraps or skirts coupled with new CT-based sizing algorithms allowed us to achieve similar or almost non-inferior short-term results with TAVI in bicuspid compared to tricuspid AS [8]. However, there is still paucity of randomized data comparing transcatheter treatment to SAVR, as those patients were consistently excluded from major randomized controlled trials (RCTs), and more importantly, very scarce data exists on the long-term performance of THVs in bicuspid native AS [10,11]. It has been shown that certain bicuspid phenotypes predict worse clinical outcomes and that the THV’s stent frames in bicuspid TAVI tend to be asymmetrical or underdeployed due to excessive calcium volumes and raphes [12,13,14]. To that end, it can be speculated that despite good acute performance, in the long run, this may induce unfavourable hemodynamics, increase leaflet strain and effectively limit the durability of the devices [15]. More high-quality, randomized controlled trials are needed to fully compare the outcomes of SAVR and TAVI for bicuspid aortic stenosis and to establish the best treatment strategy.

Small root anatomy in combination with high index THV positioning may pose coronary occlusion risk during the redo-procedure [16]. In the present registry, CT-based analysis suggested the need for coronary protection, and leaflet laceration or chimney stenting was performed in 12.5% of cases. This highlights the need for both commissural alignment during index TAVI (especially with tall frame THVs) and widespread availability of simplified devices for leaflet splitting in the near future [17,18].

In redo-TAVI, the goal is to select a bioprosthesis that provides adequate sealing and avoids patient–prosthesis mismatch, which can occur with both significantly oversizing or undersizing the valve. In our cohort, the mean redo-THV label size was 25 ± 3 mm. Such a large size of the second bioprosthesis could have resulted in a relatively low rate of postprocedural moderate/severe perivalvular leak (three (9.4%) individuals). Nevertheless, the sizing for redo-TAVI is not standardized and requires a personalized, case-based approach, considering both the patient’s anatomy and the specific features of the failed index prosthesis.

Raising another issue, regarding studies examining redo-TAVI so far, we would like to comment on short- and long-term outcomes. The first study included a cohort of 19 patients who required a redo-procedure out of 2.301 index TAVRs (0.8%) from two German centres (years 2011–2015). The incidence of 30-day and 1-year mortality was equal to 11.0% and 33.0%, while 30-day rates of strokes and PPIs were 5.0% and 11.0%, respectively [19]. Another report from a multicenter registry (years 2014–2016) that included 50 redo-TAVI patients with a median age of 78 (71–89) comes from Barbanti M et al. The mean time to the redo-procedure was 812 ± 750 days, while survival at the latest follow-up (approximately 3 years) amounted to 85.1% [20]. Landes U et al. reported data from the Redo-TAVR Registry (2019–2020) for 212 individuals, with a median follow-up time equal to 447 (95–1.091) days. The time to the redo-procedure had a very big range (IQR)—2 days to 11.6 years. The authors provide the rates of periprocedural mortality, stroke, coronary obstruction, valve mal-positioning, PPI and conversion to open surgery as equal to 0.0%, 1.4%, 0.9%, 3.3%, 9.6% and 0.4%, respectively. The 30-day survival for early and late valve dysfunction was reported as 94.6% and 98.5%, and for 1-year survival, it was 83.6% and 88.3%, respectively [3]. A study by Percy ED et al. based on the Medicare database involved 617 patients aged 76.9 ± 8.2 years who underwent redo-TAVR procedures between 2012 and 2017. The follow-up time was quite short (28 (15–43) months), while the time to the redo-procedure was 154 (58–537) days. The incidence of 30-day and 1-year mortality reached 6.0 and 22.0%, whereas 30-day stroke and PPI were 1.2 and 4.2%, respectively [21]. Another report with a maximum follow-up of 6 years comes from the TRANSIT registry (2020–2024) and encompasses 172 individuals aged 79.9 ± 7.9 years. The mean time to redo-TAVI for patients with moderate and severe patient–prosthesis mismatch (PPM) was equal to 597.5 ± 132.2 and 702 ± 946.1 days, respectively. In-hospital, 30-day and 1-year mortality reached 4.1, 7.0 and 10.0%. All strokes and MIs were reported within the first 30 days (3.5% and 1.2%) [22]. The last and the newest registries (STS/ACC TVT Registry (2011–2022)) by Makkar RR et al. included a cohort of 1.320 redo-TAVR patients aged 78 ± 9 years. The rates of procedural complications were quite low (intraprocedural mortality: 0.6%; coronary obstruction: 0.3% and conversion to open surgery: 0.5%) and similar to native-TAVR. The 30-day mortality and stroke rates were 4.7% and 2.0%, while within the first year, the rates were 17.5% and 3.2%, respectively. Unfortunately, the follow-up time, the time to the redo-procedure and indications for redo-TAVI were not reported [23].

When analyzing the short-term results of the first redo-TAVI in Poland, the rate of complications was low with two events: one major bleeding complication and one patient requiring SAVR with root replacement. There were no deaths for up to 30 days. Mortality at 1 year in the polish registry was 15.6%, which is comparable to previously published data from a large international redo-TAVI registry; also no apparent difference between the failed procedure and failed THV groups was found [3]. Surprisingly, between 30 days and 1 year, five strokes were reported (including two disabling one), representing a higher rate than that usually observed after native TAVI [24]. Obviously, the timing of these cerebrovascular events strongly suggests its non-procedural origin. Xenograft tissue and synthetic or metallic parts of THVs that are prone to stimulate a prothrombotic environment coupled with clinical risk factors (especially non-diagnosed before and immediately after redo-TAVI atrial fibrillation) are believed to be responsible for the enhanced risk of late neurological symptoms in TAVI recipients [25]. The logical assumption would be that another THV implanted inside the already existing failed one, and by adding prothrombotic material and/or creating flow-reduced niches, this could potentially lead to an increased risk of thromboembolic events [5]. It is however unknown if more aggressive routine anticoagulation in high-risk redo-TAVI patients would be beneficial in terms of leaflet thrombosis or thromboembolisation, especially since trials examining its use after native TAVI with no clinical indications (e.g., atrial fibrillation) were negative [26].

## 5. Limitations

There are several limitations associated with the current registry. First, data from early procedures were collected in a retrospective fashion with all its inherent limitations. Secondly, despite this registry being the first and largest in Poland, the available cohort of patients undergoing redo-TAVI was not big enough to allow for a more meaningful analysis, and comparisons and some observations may be incidental. Furthermore, due to the THV market share characteristics in the study period, most procedures were a combination of failed self-expanding THV treated with balloon-expanding THV. Finally, since the registry covers 11 years of redo-TAVI use in Poland, some findings may be affected by the use of older-generation devices and the evolving learning curve.

## 6. Conclusions

Initial data of redo-TAVI in Poland suggest that the procedure is safe and characterized by favourable efficacy and low rates of short-term adverse outcomes. Observed trends suggesting a high frequency of baseline native bicuspid anatomy before index TAVI and increased non-procedural late stroke occurrence after the redo-procedure must be further investigated in larger cohorts. Regarding the increasing number of redo-TAVI procedures in the world and in Poland, the preliminary results from pilot studies provide powerful data for future management and treatment of this population. However, in the end, we must emphasize that the registry is still being maintained, and the number of included patients is growing. We hope that, via infallible conducting of our database, future analyses will be more precise and that the reliability of the results will increase with the number of individuals and the duration of follow-up.

## Figures and Tables

**Table 1:** Baseline characteristics before redo-TAVI.

Age, years	75 ± 13
Male	20 (62.5)
Body mass index, kg/m^2^	28.6 ± 4.7
Body surface area, m^2^	1.90 ± 0.23
Society of Thoracic Surgeons score	6.5 ± 2.8
Diabetes mellitus	12 (37.5)
Hypertension	30 (93.8)
Chronic obstructive pulmonary disease	6 (18.8)
Atrial fibrillation	14 (43.8)
Oral anticoagulation	13 (40.6)
Prior myocardial infarction	10 (31.3)
Prior stroke or transient ischemic attack	7 (21.9)
Prior coronary artery bypass grafting	8 (25.0)
Prior percutaneous coronary intervention	16 (50.0)
New York Heart Association class III–IV	22 (68.8)
Permanent pacemaker	7 (21.9)
Chronic kidney disease	19 (59.4)
Hemodialysis	1 (3.1)
Glomerular filtration rate, mL/min/1.73 m^2^	53 ± 27
Hemoglobin, g/dL	11.6 ± 1.7

Categorical variables presented as numbers and percentages, continuous as the mean with standard deviation.

**Table 2:** Periprocedural characteristics of index TAVI.

Bicuspid anatomy (CT-based)	12 (37.5)
Type 1 (Sievers classification)	11 (91.7)
Transfemoral access	29 (90.6)
Predilatation	19 (59.3)
Postdilatation	9 (28.1)
Prosthesis type	
Self-expanding	27 (84.4)
Corevalve/Evolut R/Pro	19
Accurate Neo/Neo 2	5
Portico	2
Hydra	1
Balloon-expanding (Sapien XT/3)	4 (12.5)
Other (Lotus)	1 (3.1)
Mean prosthesis size, mm	27.7 ± 3
Discharge	
Perivalvular leak moderate/severe (echo)	6 (18.7)
Mean residual pressure gradient (echo), mmHg	12.7 (7)
Dual antiplatelet therapy	20 (62.5)
Oral anticoagulation	10 (31.3)
Single antiplatelet therapy	2 (6.2)

**Table 3:** Baseline echocardiographic parameters before redo-TAVI and mechanisms of index THV failure.

Left ventricular ejection fraction, %	52 ± 13
Aortic valve area, cm^2^	1.17 ± 0.59
Aortic valve area indexed, cm^2^/m^2^	0.53 ± 0.17
Aortic velocity, m/s	3.5 ± 1.1
Pressure gradient mean, mmHg	24 ± 21
Pressure gradient max, mmHg	42 ± 35
Stenosis	10 (31.3)
Regurgitation	19 (59.4)
Mixed	3 (9.4)
History of index THV endocarditis	4 (12.5)
History of index THV thrombosis	1 (3.1)

Categorical variables presented as numbers and percentages, continuous as the mean with standard deviation.

**Table 4:** Periprocedural characteristics of redo-TAVI.

Mean time from index TAVI, years	4.7 ± 3.5
Procedural failure * (up to 1 year)	7 (21.9)
Bicuspid as native anatomy	3 (42.8)
Transcatheter heart valve failure ** (beyond 1 year)	25 (78.1)
Bicuspid as native anatomy	9 (36)
Transfemoral access	32 (100)
Prosthesis type	
Balloon-expanding	24 (75)
Sapien 3/Ultra	23
Myval	1
Self-expanding	8 (25)
Evolut R/Pro	7
Portico	1
Mean prosthesis size, mm	25.7 ± 3
Balloon-expandable in self-expandable	22 (68.7)
Self-expandable in balloon-expandable	6 (18.7)
Self-expandable in self-expandable	3 (9.4)
Balloon-expandable in balloon-expandable	1 (3.1)
Predilatation	7 (21.8)
Postdilatation	11 (34.4)
Coronary protection	4 (12.5)
Chimney stenting	2 (50)
Leaflet laceration (BASILICA)	2 (50)
Discharge	
Perivalvular leak moderate/severe (echo)	3 (9.4)
Mean residual pressure gradient (echo), mmHg	13.1 ± 5.5
Mean residual gradient >20 mmHg	3 (9.4)
NYHA I-II	30 (93.7)
Dual antiplatelet therapy	5 (15.6)
Oral anticoagulation	15 (46.9)
Single antiplatelet therapy	12 (37.5)

*: valve mal-position or mal-sizing during the index TAVI procedure; **: valve structural deterioration (including thrombosis and endocarditis).

**Table 5:** Key outcomes after redo-TAVI up to 1 year.

All-cause death *	5 (15.6)
Cardiovascular death *	3 (9.4)
Stroke *	5 (15.6)
Need for surgical aortic valve replacement	1 (3.1)
Pacemaker implantation	0 (0)
New left bundle branch block	3 (9.4)
Life-threatening bleeding/vascular complication	1 (3.1)
Coronary obstruction	0 (0)
Hospitalization due to heart failure	3 (9.4)
Valve thrombosis	1 (3.1)
Valve endocarditis **	1 (3.1)

* All deaths and neurological events occurred after 30 days; ** additionally 1 endocarditis event occurred on the native mitral valve.

## Data Availability

The original contributions presented in this study are included in the article. Further inquiries can be directed to the corresponding author.
